# Central low-grade osteosarcoma with an unusual localization in the diaphysis of a 12-year old patient

**DOI:** 10.2478/raon-2013-0015

**Published:** 2013-05-21

**Authors:** Magdalena Maria Gilg, Bernadette Liegl, Christine Wibmer, Werner Maurer-Ertl, Andreas Leithner

**Affiliations:** 1 Department of Orthopedics and Orthopedic Surgery, Graz, Austria; 2 Institute of Pathology, Medical University of Graz, Graz, Austria

**Keywords:** fibrous dysplasia, low-grade osteosarcoma, diaphysis

## Abstract

**Background:**

Low-grade central osteosarcoma is a very rare subtype of osteosarcoma with a predilection for the metaphysis of long bones and a peak incidence in the 3^rd^ decade of life. Absence of specific clinical symptoms and a good prognosis after wide resection are the characteristics of this entity. Chemotherapy is not indicated in this highly differentiated tumour.

**Case report:**

A 12-year old girl presented with limping, swelling and pain in the mid of the left femur. Radiography showed a 12 cm long intraosseous expansion with lamellated periosteal reaction and contrast medium enhancement in MRI. Although radiology led to the differential diagnoses of Ewing’s sarcoma, osteomyelitis and fibrous dysplasia, the histological specimen showed a hyopocellular spindle-cell proliferation arranged in fascicles with mild cytologic atypia and only single mitotic figures. In synopsis with radiology the diagnosis of low-grade central osteosarcoma was made and confirmed by reference pathology. The tumour was resected with wide margins and reconstruction was performed with a vascularized fibula, a homologous allograft and a plate. Staging was negative for recurrence and metastasis at a follow-up of 16 months.

**Conclusions:**

Low-grade osteosarcoma accounts for only 1% of all osteosarcomas with a peak incidence in the 3^rd^ decade. The diaphyseal localization and the young age make this case special. To achieve the correct diagnosis of this rare low-grade entity and thereby the adequate treatment, despite a wide range of differential diagnoses, a multidisciplinary approach is essential.

## Introduction

Although osteosarcomas are the most frequent primary bone tumours, they account for less than 1% of all cancers diagnosed in the United States.^1^ Low-grade central osteosarcoma (LGCOS) accounts for only 1.2% of all osteosarcomas.^2^ The aetiology of LGCOS is not fully elucidated, except for Ragazzini *et al*. reporting that SAS, MDM2 and CDK4 genes may be involved in tumourogenesis and progression of this tumour.^3^ This very rare bone tumour has a good prognosis when the excision with wide margins can be achieved. Therapy of choice is the complete excision with wide margins to avoid the local recurrence of the tumour. Chemo- or radiotherapy are not indicated. When treated correctly LGCOS shows a good prognosis with 5- and 10-year survival rates of 90% and 85%. Dedifferentiation of LGCOS to high-grade osteosarcoma can occur in 15% of recurrent tumours including the possibility of metastases. Thus, recurrence is not seen in wide resections, but local excision is almost always followed by recurrence.^4^–^7^ Herein we describe the clinical, radiological and pathologic features of a 12-year old patient diagnosed with LGCOS localized in the diaphysis of the left femur.

## Case report

A 12-year old girl was referred to an orthopaedic tumour centre due to the increasing pain in the left diaphyseal femur when walking. The pain started 3 days prior to the presentation and did not persist at night or at rest. Additionally the patient herself detected a mass in the left femur. The clinical examination revealed a visible and palpable swelling of the femur. On exertion of pressure it was painful. The surrounding skin and the remaining examination were normal. There was no history of weight loss, fever, chills, fatigue or exhaustion. The patient had no relevant past medical history. Routine laboratory tests were within normal range.

**FIGURE 1A,B.**
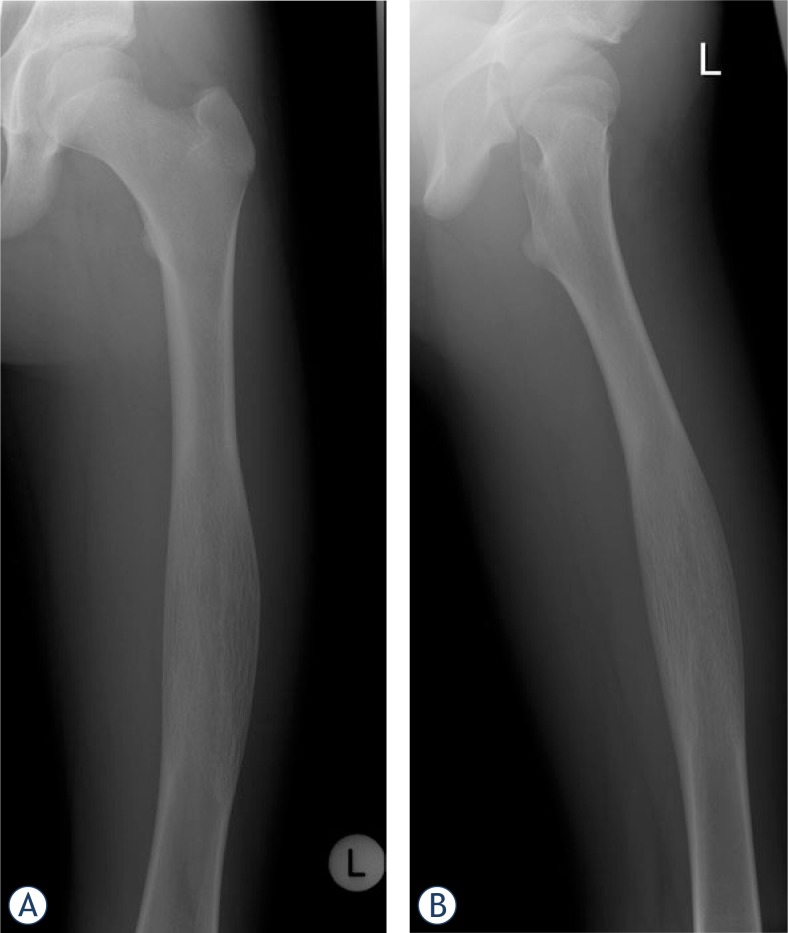
X-ray shows a 12 cm intraosseous expansion and lamellated periosteal reaction in the diaphysis of the left femur (anterio-posterior, lateral).

Radiography showed an osseous expansion with lamellated periosteal reaction in the diaphysis of the left femur (Figure 1A,B). The MR scan showed in T1-weighed imaging in the mid of the left femur with contrast medium enhancement, lesion in the mid of the left femur. Cortical destruction was present. The size of the lesion was 12 cm (cranio-caudal) × 3,5 cm (sagittal) × 4 cm (transversal) (Figure 2A). Whole body bone scan showed the increased uptake of the tracer in the diaphysis of the left femur, but no evidence of further lesions (Figure 2B). Staging was negative. Radiological differential diagnoses included fibrous dysplasia, Ewing’s sarcoma and chronic osteomyelitis. Incisional biopsy was conducted from the lateral side. The biopsy specimen showed a hypocellular spindle-cell proliferation arranged in fascicles with mild cytologic atypia and only single mitotic figures. The tumour proliferation permeated surrounding bone structures and entrapped bony trabeculae (Figure 3A,B). Diagnosis of a low-grade central osteosarcoma of the diaphysis was made.

**FIGURE 4:**
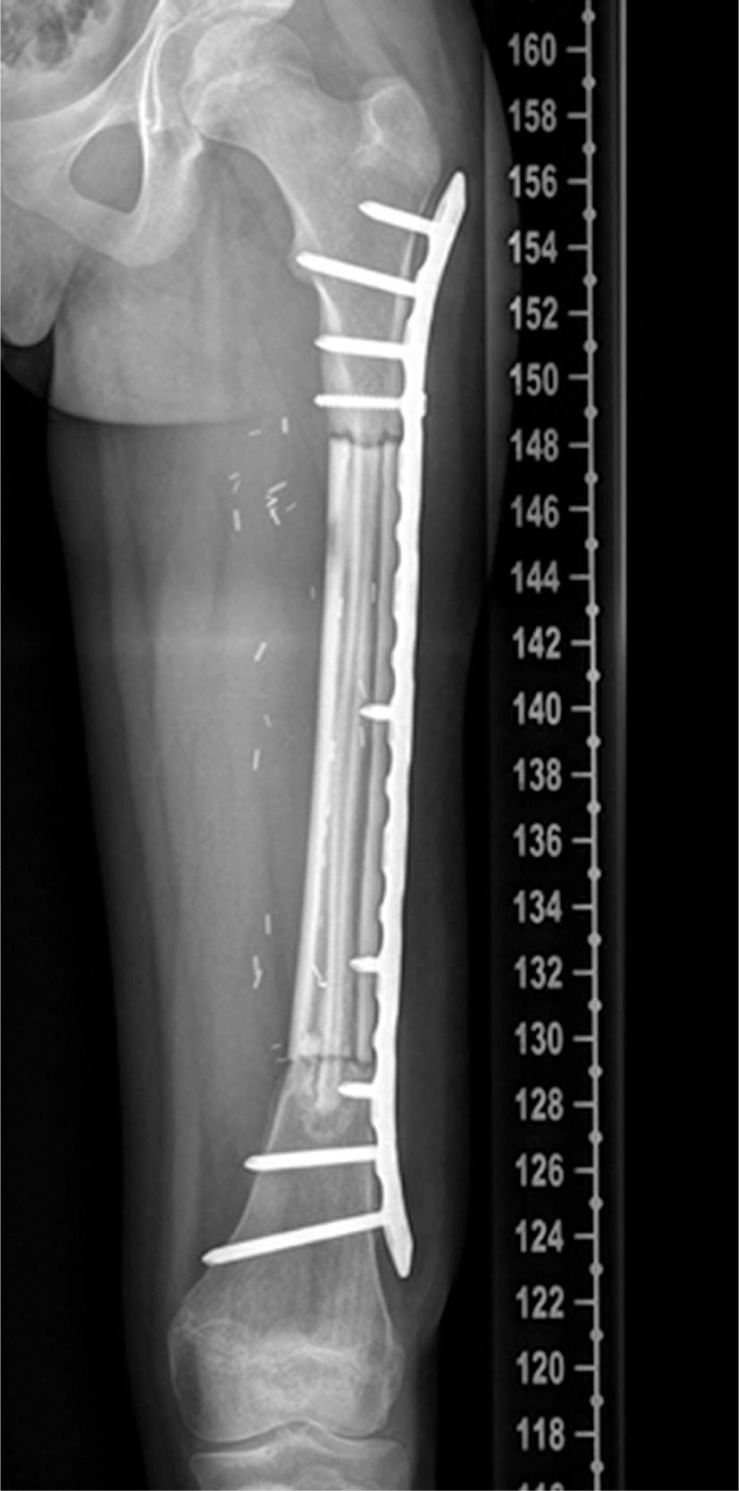
The X- ray shows the reconstruction of the femur with a vascularized fibula, a homologous allograft and a plate (11 months postoperatively).

The treatment included a resection with wide margins and a reconstruction of the femur with a contralateral vascularized fibula, a homologous allograft and a plate (Figure 4). Fifteen months after reconstruction the patient had a bike accident in which the plate broke and revision surgery had to be performed. Staging, including ultrasound of the abdomen and retroperitoneum as well as CT scans of the chest, were normal on follow- up.

## Discussion

LGCOS was first described by Unni *et al*. in 1977.^4^ Up to now literature on LGCOS is restricted to a very limited number of publications with very few original articles focusing on LGCOS within the past 15 years. Regarding epidemiologic features the majority of patients were aged between 18 and 45 years and therefore slightly older than patients with conventional high-grade osteosarcoma.^7^ Our patient belonged to a minority since only 7–21% of patients in other studies were younger than 18 years.^5^,^7^ There is only one patient described in literature younger than 12 years.^7^ Male to female ratio is almost equal in contrast to conventional osteosarcoma slightly predominating the male gender.^2^,^7^

LGCOS is typically localized in the long bones with a predilection for the lower limb, especially the femur is most frequently affected.^4^,^5^,^7^ Uncommon areas of localization known to literature are flat bones, skull, facial bones and small bones of the hands and feet.^8^ Within the long bone the metaphysis or the diametaphysis are affected in 84%, the remainder is diaphyseal.^2^,^7^ There are no characteristic symptoms of this tumour, since only pain or swelling or none of the latter may be present. If present the duration of symptoms is about 5–12 months and therefore longer than in conventional osteosarcoma.^2^,^5^

Macroscopically, LGCOS is a well demarcated and large tumour with 2–25 cm in greatest dimension. The appearance of the tumour is variable from firm and gritty to white- fibrous and rubbery tissue. Hemorrhagic areas may be present as well as extension into the adjacent soft tissue.^2^,^5^,^7^ In LGCOS there is no homogenous histological pattern of growth as in high-grade osteosarcoma. The tumour proliferation permeates surrounding bone structures and entraps bony trabeculae. LGCOS consists of spindle-cells exhibiting only scarce cytologic atypia and few mitotic figures. This pauci-cellular lesion infiltrates between bone trabeculae. The amount of osteoid or bone produced by the cells is variable. The matrix consists of heavy and irregular bone trabeculae. When sectioning the tumour, woven microtrabeculae of bone in a moderately cellular fibrous stroma can be detected. The lesion interfaces with the normal bone, since fibrous tissue within the Havers-Canals or between mature cancellous trabeculae can be found.^2^,^5^,^7^–^9^

Radiographs show a variable appearance in LGCOS, mimicking benign lesions, such as fibrous dysplasia (FD). Andresen et al. have described four different radiographic patterns of LGCOS: lytic with varying amounts of thick and coarse trabeculation, predominantly lytic with few thin, incomplete trabecula, densly sclerotic and mixed sclerotic and lytic. MRI or CT scans are mandatory to detect features of aggressiveness, such as cortical disruption and intramedullary or soft tissue expansion. Thus MRI and CT scans are the modality of choice, to differentiate between benign lesions and low-grade malignancies.^10^

As differential diagnoses benign and low- grade malignant lesions have to be considered. LGCOS is initially often misdiagnosed as FD, due to similar histological and radiological findings.^10^ Histological differences of fibrous dysplasia are lack of trabecular bone formation, no permeative pattern or cytologic atypia. When histology is inconsistent, radiology can provide the important clue to the diagnosis as, in contrast to LGCOS, cortical disruption and soft tissue expansion are absent in FD.^2^,^4^,^7^ In case of inconsistent histological and radiological findings, screening for GNAS1 mutation can be pursued. GNAS1 mutation can be detected in the course of McCune-Albright syndrome manifesting with fibrous dysplasia and endocrine dysfunctions.^11^ The recent research suggests immunhistochemistry of MDM 2 and CDK 4 as a sensitive marker for LGCOS. In 90% of LGCOS cases immunochemistry was positive for MDM2 and CDK4, but never in benign lesions.^12^

Desmoplastic fibromas, low-grade fibrosarcomas and parosteal osteosarcoma are differential diagnoses for low-grade malignant tumours. Parosteal osteosarcoma can be ruled out by its location, since this tumour does not infiltrate into the medullary cavity. Both desmoplastic and low- grade fibrosarcoma can radiologically resemble LGCOS, but there’s a lack of bone formation in these entities.^7^

## Conclusions

Considering the patient’s age and the diaphyseal location in the presented case, these features are very uncommon within an already extremely rare tumour entity. A constellation like this makes it difficult to obtain a correct diagnosis, which can only be achieved by a multidisciplinary approach, including radiology, pathology and orthopaedics. When misdiagnosed or mistreated the patient is set at risk to develop high-grade osteosarcoma and metastasis. Unnecessary exposition to chemo- or radiotherapy has to be avoided. Therefore, one should be aware of this rare subtype of osteosarcoma and LGCOS should be considered as a differential diagnosis.

## Figures and Tables

**FIGURE 2A:**
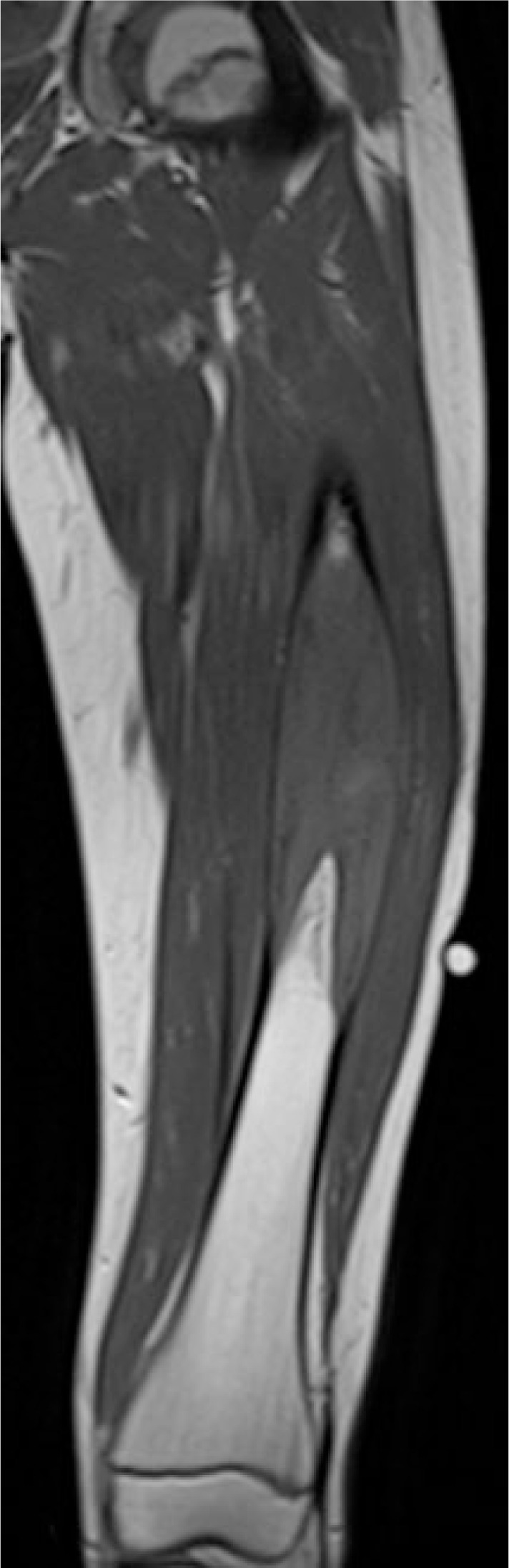
T1 weighed MR imaging detects a hyperintense, contrast medium enhanced, lesion in the mid of the left femur. Cortical destruction can be seen. The biopsy tract can be seen on the lateral side.

**FIGURE 2B:**
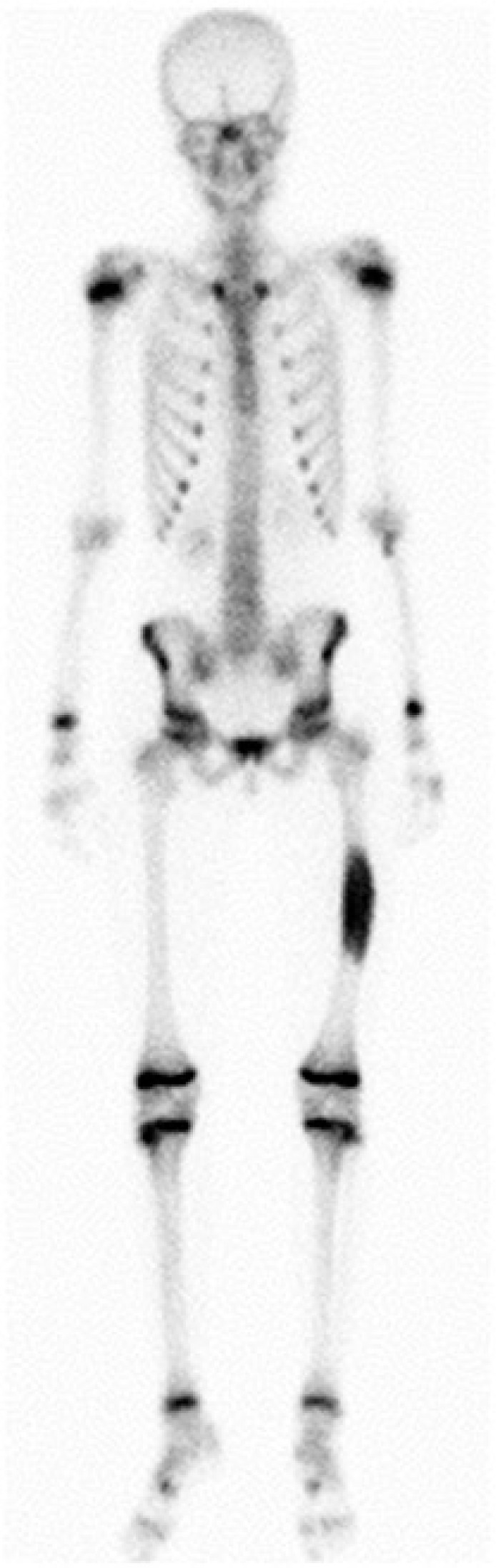
Whole body bone scan showed increased uptake of the tracer in the diaphysis of the left femur (Tc-99m-3 phases bone scintigraphy).

**FIGURE 3A:**
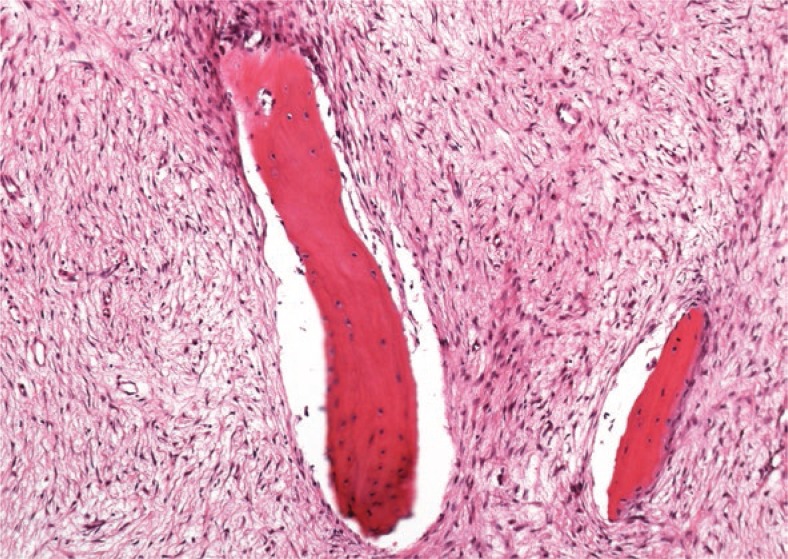
The tumour is composed of a well differentiated fibroblastic component entrapping bony *trabeculae*. The spindle cells are set in a collagenous matrix (H&E stain).

**FIGURE 3B:**
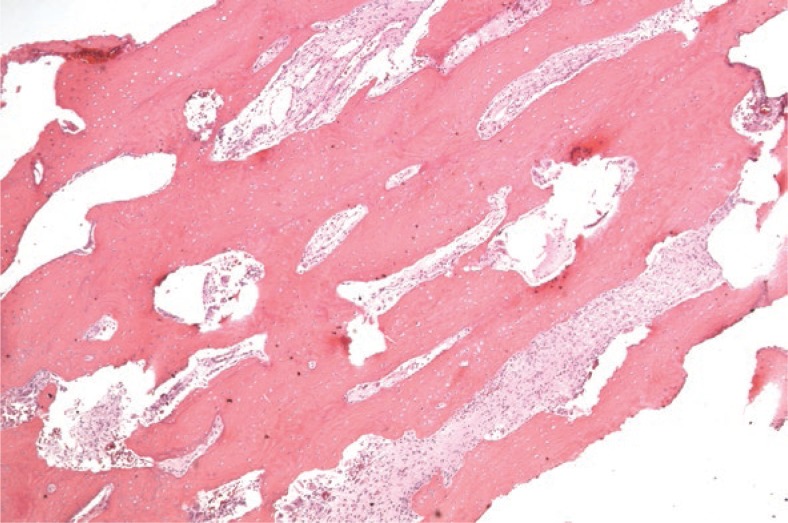
On low power magnification long parallel seams of bone are surrounded by a hypocellular spindle cell stroma (H&E stain).
